# Associations between diabetes mellitus and pulmonary hypertension in chronic respiratory disease patients

**DOI:** 10.1371/journal.pone.0205008

**Published:** 2018-10-09

**Authors:** Tomoko Takahashi, Akiomi Yoshihisa, Koichi Sugimoto, Tetsuro Yokokawa, Tomofumi Misaka, Takashi Kaneshiro, Masayoshi Oikawa, Atsushi Kobayashi, Kazuhiko Nakazato, Takafumi Ishida, Yasuchika Takeishi

**Affiliations:** 1 Clinical Research Center, Fukushima Medical University Hospital, Fukushima, Japan; 2 Department of Advanced Cardiac Therapeutics, Fukushima Medical University, Fukushima, Japan; 3 Department of Pulmonary Hypertension, Fukushima Medical University, Fukushima, Japan; 4 Department of Arrhythmia and Cardiac Pacing, Fukushima Medical University, Fukushima, Japan; 5 Department of Cardiovascular Medicine, Fukushima Medical University, Fukushima, Japan; Kurume University School of Medicine, JAPAN

## Abstract

**Background:**

Pulmonary hypertension (PH) is a common complication of chronic respiratory disease. Recent studies have reported diabetes mellitus (DM) to be a poor prognostic factor in patients with chronic respiratory disease, including chronic obstructive pulmonary disease or interstitial pneumoniae. However, the association between DM and PH in chronic respiratory disease remains unclear. In this study, we aimed to investigate whether DM is a predictor of PH in patients with chronic respiratory disease.

**Methods:**

We prospectively analyzed 386 patients in our hospital with chronic respiratory disease. An echocardiographic pressure gradient between the right atrium and the right ventricle of **≥** 40 mmHg was defined as PH. We compared the clinical characteristics and impact of DM between chronic respiratory disease patients with and those without PH.

**Results:**

Of the 386 patients, 42 (10.9%) were diagnosed as having PH. The PH group had higher modified medical research council (mMRC) grade and complication rate of DM, but not hypertension and hyperlipidemia, when compared to the non-PH group. Multivariable logistic regression analysis revealed that mMRC scale (odds ratio 1.702, 95% confidence interval, 1.297 to 2.232, P < 0.001) and presence of DM (odd ratio 2.935, 95% confidence interval, 1.505 to 5.725, P = 0.002) were associated with PH in chronic respiratory disease patients.

**Conclusion:**

DM is potentially associated with PH and is an independent factor for prediction of PH in patients with chronic respiratory disease.

## Introduction

Pulmonary hypertension (PH) due to chronic respiratory disease, such as chronic obstructive pulmonary disease (COPD) and/or interstitial pneumonia, is classified as group 3 PH by the World Health Organization and has been reported to be a common form of PH cases [1].

Various degrees of PH occur in severe respiratory disease. The destruction, occlusion, and narrowing of the pulmonary vascular bed due to respiratory disease decrease the total cross-sectional area of the pulmonary vascular bed and increase pulmonary vascular resistance [2, 3]. In addition, hypoxemia and hypercapnia due to impaired gas exchange readily cause pulmonary vasoconstriction [3], and further, compensatory polycythemia due to hypoxemia leads to increased blood viscosity and increases pulmonary vascular resistance [4]. While increases in pulmonary arterial pressure are typically relatively mild to moderate, the prognosis of group 3 PH is poor compared to that of PH due to other causes [2, 5].

The PH and right heart failure symptoms are non-specific [3], and it is important to consider PH when a patient shows symptoms, such as shortness of breath, unexplained by respiratory function tests. Measurement of the tricuspid regurgitation pressure gradient (TR-PG) by transthoracic echocardiography is the most useful method among noninvasive examinations to check for PH [1]. In addition, right heart catheterization (RHC), which is essential for a definite diagnosis of PH (defined as mean pulmonary arterial pressure > 20 mmHg and pulmonary vascular resistance (PVR) ≥ 3 WoodU), is invasive and thus difficult to perform on all patients with respiratory disease. According to the European Society of Cardiology (ESC) and the European Respiratory Society (ERS) guidelines of 2015, RHC is not recommended for suspected PH with lung disease unless therapeutic consequences are expected [1]. Therefore, it is important to determine the clinical factors that predict PH, so that high-risk patients can be identified.

Recently, associations between metabolic disorders and PH have been reported [6, 7]. In addition, Ho et al. have reported that diabetes mellitus (DM) is a vital factor in the prognosis of patients with COPD [8].

In this study, we examined the associations between DM and PH in patients with chronic respiratory disease.

## Methods

### Subjects

This was a prospective study, which included 386 consecutive patients who had interstitial pneumonia or COPD diagnosed by expert pulmonologists and who underwent transthoracic echocardiography performed by expert cardiologists in our hospital between 2006 and 2016. COPD was diagnosed when patients met forced expiratory volume in 1 s (FEV_1_)/forced vital capacity (FVC) < 70% by spirometry as Global Initiative for Chronic Obstructive Lung Disease criteria [9]. On the basis of the patient’s clinical history; computed tomography scans of the chest; and surfactant protein-A, protein-D, and KL-6 levels, diagnoses of interstitial pneumoniae was comprehensively made by expert pulmonologists in our hospital according to the American Thoracic Society and ERS guidelines [10]. Interstitial pneumoniae included (i) idiopathic, (ii) connective-tissue-disease-associated, and (iii) unclassified interstitial pneumoniae. Hypertension was defined as the recent use of antihypertensive drugs, systolic blood pressure of ≥ 140 mmHg, and/or diastolic blood pressure of ≥ 90 mmHg [11]. DM was defined as the recent use of antidiabetic dugs, a fasting glucose ≥ 126 mg/dL, a casual glucose ≥ 200 mg/dL, and/or HbA1c ≥ 6.5% (National Glycohemoglobin Standardization Program) [12]. Dyslipidemia was defined as the recent use of cholesterol-lowering drugs, triglycerides ≥ 150 mg/dL, low-density lipoprotein cholesterol ≥ 140 mg/dL, and/or high-density lipoprotein cholesterol < 40 mg/dL [13]. A smoker was defined as a person who had smoked any cigarette in the past 3 months. Patients with acute heart failure or acute respiratory failure were excluded. Echocardiographic parameters and laboratory data were obtained from medical records. Dyspnea was assessed using modified Medical Research Council (mMRC) scale because of its simplicity and versatility [14]. The clinical research protocol was approved by the ethical review board of Fukushima Medical University in accordance with the Declaration of Helsinki.

### Pulmonary function test

The pulmonary function test was performed using a spirometer (CHESTAC-8900, Chest, Tokyo, Japan) when the patients were in a relatively stable phase or during euvolumic phase. Spirometry was defined using standard indices: FEV_1_, FVC, FEV_1_/FVC, vital capacity, and percentage vital capacity [15].

### Echocardiography

**Fig 1:**
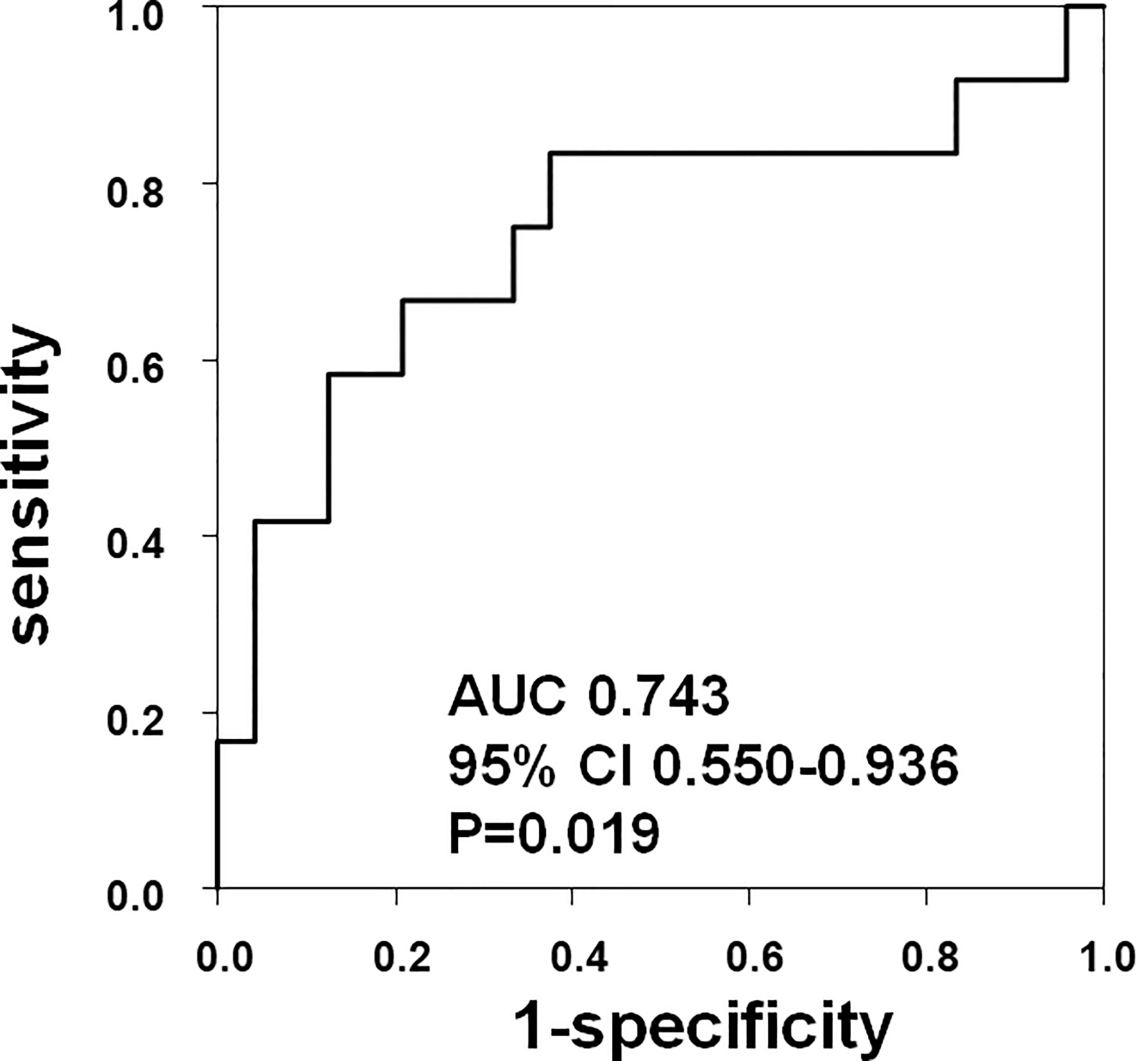
Receiver operating characteristic (ROC) curve for tricuspid regurgitation pressure gradient (TR-PG) for prediction of pulmonary hypertension (PH) in patients with chronic respiratory disease. As per the ROC curve, the sensitivity and specificity to detect PH defined by right heart catheterization were 58.3% and 87.5%, respectively, using the cut-off value as TR-PG = 40 mmHg. AUC, area under the curve; CI, confidence interval.

Transthoracic echocardiography was performed by experienced echocardiographers using standard techniques [16]. TR-PG, right ventricular fractional area change (RV-FAC), left ventricular ejection fraction (LVEF), and ratio of mitral peak early filling velocity (E) to early mitral annular velocity (e’) (E/e’) were measured as indices of echocardiography [15]. Since no clear TR-PG cut-off values are available for diagnosing PH in respiratory disease patients [17], we defined PH as TR-PG ≥ 40 mmHg according to a previous report [18]. Receiver operating characteristic (ROC) curve demonstrated that sensitivity and specificity to detect PH based on RHC were 58.3% and 87.5%, respectively, with the above mentioned cut-off value, TR-PG 40 mmHg (Fig 1). All recordings were done using an ultrasound system (ACUSON Sequoia, Siemens Medical Solutions, Mountain View, CA, USA).

### Hemodynamics measurements

All catheterizations were performed within 7 days after echocardiography in a resting supine position under fluoroscopic guidance. Pulmonary arterial pressure, pulmonary capillary wedge pressure, and cardiac output were measured using a 7F Swan-Ganz catheter (Edwards Lifesciences, Irvine, CA, USA). We mainly used the thermo dilution method for the measurement of cardiac output, however, for cases of advanced tricuspid regurgitation, we used the Fick method. PVR was calculated using the conventional formula.

### Statistical analysis

Normally distributed data are presented as mean ± SD, and categorical variables are expressed as numbers and percentages. The data were compared using the Student t-test, and the chi-square test was used for categorical variables. We performed multiple logistic regression analysis allowing for interaction between the presence of PH and the following clinical confounding factors: age, sex, body mass index, mMRC scale, hypertension, DM, dyslipidemia and smoking. These analyses were performed using a statistical software package (SPSS ver. 24.0, IBM, Armonk, NY, USA).

## Results

Patient characteristics are shown in Table 1. Of the 386 patients with respiratory disease (131 in COPD and 255 in interstitial pneumoniae), 42 (10.9%) had PH. In addition, 127 of the patients (32.9%) had concomitant DM.

**Table 1:** Comparisons of clinical features.

	Non-PH (TR-PG < 40 mmHg) n = 344	PH (TR-PG ≥ 40 mmHg) n = 42	*P* value
Age (years)	68.9 ± 11.2	71.4 ± 12.9	0.176
Male sex (n, %)	238 (69.2)	30 (71.4)	0.860
Body mass index (kg/m^2^)	22.9 ± 4.3	22.1 ± 5.1	0.265
mMRC scale	1.6 ± 1.2	2.4 ± 1.4	<0.001
COPD (n, %)	114 (33.1)	17 (40.5)	0.343
IP (n, %)	230 (66.9)	25 (59.5)	0.343
Co-morbidity			
Hypertension (n, %)	176 (51.2)	25 (59.4)	0.330
Diabetes mellitus (n, %)	104 (30.2)	23 (54.8)	0.003
Dyslipidemia (n, %)	144 (41.9)	20 (47.6)	0.511
Smoking (n, %)	235 (68.3)	31 (73.8)	0.597
Echocardiography			
TR-PG (mmHg)	23.9 ± 7.8	53.0 ± 16.8	<0.001
LVEF (%)	63.1 ± 8.5	62.6 ± 14.2	0.227
RV-FAC (%)	45.0 ± 13.6	38.6 ± 17.7	0.041
E/e’	9.8 ± 6.8	11.6 ± 5.2	0.164
Pulmonary function test			
VC (L)	2.6 ± 0.9	2.3 ± 0.9	0.120
%VC (%)	81.1 ± 21.9	77.4 ± 23.5	0.309
FVC (L)	2.5 ± 0.9	2.3 ± 0.9	0.069
FEV_1_ (L)	1.9 ± 0.7	1.7 ± 0.7	0.075
FEV_1_/FVC (%)	75.1 ± 15.8	75.0 ± 17.1	0.977
%FEV_1_ (%)	79.8 ± 26.5	77.0 ± 30.0	0.533
Laboratory data			
KL-6 (U/mL)	1799.8 ± 1511.4	1450.0 ± 1105.1	0.227
SP-D (ng/mL)	306.4 ± 248.1	259.5 ± 205.6	0.328
SP-A (ng/mL)	100.0 ± 62.8	95.2 ± 65.3	0.700
HbA1c (%)	5.9 ± 0.8	5.9 ± 0.7	0.685

E/e’, ratio of mitral peak early filling velocity to early mitral annular velocity; FEV_1_, forced expiratory volume in 1 s; FVC, forced vital capacity; LVEF, left ventricular ejection fraction; mMRC, modified Medical Research Council; RV-FAC, right ventricular fractional area change; SP-A, surfactant protein-A; SP-D, surfactant protein-D;.TR-PG, tricuspid regurgitation pressure gradient; VC, vital capacity.

The subjects in the PH group had higher scores on the mMRC scale than those in the non-PH group. Furthermore, the prevalence of DM in the PH group was significantly higher than that in the non-PH group (54.8% vs. 30.2%, P = 0.003). In contrast, the prevalence of hypertension and dyslipidemia did not significantly differ between the two groups. Regarding echocardiographic parameters, RV-FAC was significantly lower in the PH group than in the non-PH group (38.6 ± 17.7% vs. 45.0 ± 13.6%, P = 0.041). In contrast, LVEF and E/e’ did not differ between the two groups. Additionally, there were no significant differences between the pulmonary function tests results and laboratory data of the two groups.

In the univariate logistic regression analysis, higher mMRC scale and presence of DM were found to be associated with PH complication (odds ratio 1.680, 95% confidence interval, 1.286 to 2.195 [mMRC scale], P < 0.001; odds ratio 2.794, 95% confidence interval, 1.459 to 5.350 [DM], P = 0.002). In the multivariate logistic regression analysis, DM and mMRC scale were shown to be independent predictors of PH complication (odds ratio 1.702, 95% confidence interval, 1.297 to 2.232 [mMRC scale], P < 0.001; odds ratio 2.935, 95% confidence interval, 1.505 to 5.725 [DM], P = 0.002). (Table 2)

**Table 2:** Logistic analysis for potential predictors of PH.

	Univariate			Multivariate		
Variables	Odds ratio	95% CI	*P* value	Odds ratio	95% CI	*P* value
Age	1.023	0.990–1.056	0.177			
Male sex	0.898	0.443–1.822	0.766			
Body mass index	0.956	0.885–1.034	0.264			
mMRC scale	1.680	1.286–2.195	<0.001	1.702	1.297–2.232	<0.001
Hypertension	1.404	0.732–2.693	0.308			
Diabetes mellitus	2.794	1.459–5.350	0.002	2.935	1.505–5.725	0.002
Dyslipidemia	1.263	0.664–2.400	0.477			
Smoking	1.307	0.633–2.697	0.469			

mMRC, modified Medical Research Council

We verified that DM predicts PH in patients who underwent RHC. In our study, of the 45 patients on whom we performed RHC, 13 had PH. The number of patients with DM was significantly higher in the PH group than in the non-PH group (69.2% vs. 31.3%, P = 0.019; Table 3). Univariate analysis revealed that only DM is associated with PH in chronic respiratory disease (odds ratio 4.950, 95% confidence interval, 1.227 to 19.973; P = 0.025; Table 4).

**Table 3:** Comparison of clinical features of patients who underwent right heart catheterization.

	Non-PH n = 32	PH (mPAP > 20 and PVR ≥ 3WU) n = 13	*P* value
Age (years)	73.5 ± 10.9	70.4 ± 13.8	0.421
Male sex (n, %)	29 (90.6)	9 (69.2)	0.073
Body mass index (kg/m^2^)	23.1 ± 4.7	21.3 ± 5.0	0.282
mMRC scale	1.8 ± 1.2	2.2 ± 1.4	0.275
Co-morbidity			
Hypertension (n, %)	22 (68.8)	7 (53.8)	0.344
Diabetes mellitus (n, %)	10 (31.3)	9 (69.2)	0.019
Dyslipidemia (n, %)	17 (53.1)	8 (61.5)	0.607
Smoking (n, %)	25 (78.1)	10 (76.9)	0.930
Echocardiography			
TR-PG (mmHg)	25.4 ± 4.5	42.0 ± 20.3	0.005
LVEF (%)	65.6 ± 10.1	71.8 ± 5.1	0.148
RV-FAC (%)	53.8 ± 16.7	43.1 ± 14.1	0.112
E/è	11.6 ± 4.9	10.8 ± 5.6	0.643
Hemodynamics			
Systolic PAP (mmHg)	27.9 ± 6.1	41.6 ± 10.8	<0.001
Diastolic PAP (mmHg)	12.1 ± 4.0	17.8 ± 7.4	0.002
Mean PAP (mmHg)	18.1 ± 4.3	26.8 ± 7.6	<0.001
Mean PcwP (mmHg)	8.8 ± 3.4	8.2 ± 4.2	0.594
Cardiac output (L/min)	4.7 ± 1.4	3.7 ± 1.1	0.028
PVR (Wood unit)	2.1 ± 0.8	5.2 ± 2.1	<0.001
Pulmonary function test			
VC (L)	2.5 ± 0.8	2.1 ± 0.8	0.122
%VC (%)	77.8 ± 19.7	67.4 ± 21.5	0.131
FVC (L)	2.4 ± 0.8	2.0 ± 0.9	0.094
FEV_1_ (L)	1.7 ± 0.6	1.6 ± 0.8	0.525
FEV_1_/FVC (%)	70.1 ± 14.8	78.0 ± 19.1	0.150
%FEV_1_ (%)	72.6 ± 22.7	66.7 ± 30.4	0.485
Laboratory data			
KL-6 (U/mL)	1305.3 ± 1006.8	1277.8 ± 958.5	0.945
SP-D (ng/mL)	284.9 ± 238.8	285.0 ± 244.0	1.000
SP-A (ng/mL)	99.2 ± 73.7	104.0 ± 75.7	0.876
HbA1c (%)	5.9 ± 1.2	6.3 ± 1.0	0.289

E/e’, ratio of mitral peak early filling velocity to early mitral annular velocity; FEV_1_, forced expiratory volume in 1 s; FVC, forced vital capacity; LVEF, left ventricular ejection fraction; mMRC, modified Medical Research Council; PAP, pulmonary arterial pressure; P_CW_P, pulmonary capillary wedge pressure; PVR, pulmonary vascular resistance; RV-FAC, right ventricular fractional area change; SP-A, surfactant protein-A; SP-D, surfactant protein-D;.TR-PG, tricuspid regurgitation pressure gradient; VC, vital capacity.

**Table 4:** Logistic analysis of potential predictors of PH in patients who underwent right heart catheterization.

	Univariate		
Variables	Odds ratio	95% CI	*P* value
Age	0.979	0.929–1.031	0.424
Male sex	4.296	0.806–22.900	0.088
Body mass index	0.918	0.786–1.072	0.279
mMRC scale	1.346	0.794–2.281	0.270
Hypertension	0.530	0.141–1.989	0.347
Diabetes mellitus	4.950	1.227–19.973	0.025
Dyslipidemia	1.412	0.379–5.261	0.607
Smoking	0.933	0.200–4.347	0.930

mMRC, modified Medical Research Council

## Discussion

In the current study, DM was demonstrated to be an independent factor for predicting PH complication in patients with respiratory diseases, such as COPD and interstitial pneumonia.

Pan et al. reported that elevation of right ventricular pressure due to hypoxia exposure was more pronounced in DM model mice than in their control group, and that increased production of reactive oxygen species and decreased superoxide dismutase were found in the pulmonary arterial endothelial cells of DM mice exposed to hypoxia [19]. Moral-Sanz et al. also reported that hypoxia and DM independently induce pulmonary arterial remodeling and downregulate bone morphogenic protein receptor type 2 [20]. These results provided a basis for DM to predict PH in chronic respiratory disease patients with DM. A few other studies demonstrated that PH is associated with peroxisome proliferator–activated receptor-γ signaling, which is involved in insulin resistance [21, 22], suggesting a possible mechanism by which DM predicts PH.

Several studies on humans have demonstrated the association of PH with metabolic syndrome, insulin resistance, and dyslipidemia. For example, Zamanian et al. published the first clinical report of an association between insulin resistance and PH [23]. Pugh et al. reported that unrecognized glucose intolerance, as assessed by HbA1c, is common in pulmonary arterial hypertension [24]. In addition, Abernethy et al. showed that DM is an independent predictor for the prognosis of PH patients [25]. Furthermore, Grinnan et al. described that DM is an independent predictor for PH development, even when the other components of metabolic syndrome are controlled (odds ratio = 1.53; P < 0.001) [26]. However, most of the patients in these studies were in group 1 (pulmonary arterial hypertension) and patients in group 3 were rarely included, whereas all our study subjects had chronic respiratory disease patients.

Although several studies have shown that DM affects the prognosis of both COPD and interstitial pneumoniae, the involvement of PH is still unclear. Makarevich et al. showed that PH is more severe in patients with COPD with type 2 DM, but not type 1 DM, compared to patients with only COPD [27]. Since patients with type 1 DM did not exist unexpectedly in our study, the difference between type 1 and type 2 DM with regard to the occurrence of PH in chronic respiratory disease remained unclear.

In addition, it has been reported that, of patients with COPD, 16% already had DM at the time of enrollment, and an additional 19% developed DM during the follow-up period [8]. Abernethy et al. reported a rate of DM occurrence in patients with group 3 PH of 29.8% [25]. The findings of those studies, taken together with the results of the current study, indicate that DM is also a common disease in respiratory disease patients. Further hormonal study into insulin, glucagon and corticoid in chronic respiratory disease with DM is required to confirm this finding.

### Study limitations

This study has several limitations. First, the study involved a relatively small number of samples, all of which were from a single institution. Although we performed multiple logistic regression analyses to adjust possible confounding factors as much as possible, we could not rule out residual confounding factors from unknown or unmeasured variables. Second, we were unable to show the association between PH and sleep-disordered breathing because few patients underwent sleep examinations. Third, the PH and non-PH groups were classified on the basis of TR-PG values using echocardiography. RHC is a more desirable method of defining PH; however, we could perform RHC on only 45 patients. Although overestimation and underestimation are both possibilities with echocardiography, it still seems to be the most appropriate method for screening PH in respiratory disease.

Thus, future studies involving multiple institutions and research results of right heart catheterization are needed to validate our findings in this study.

## Conclusion

We reported here the potential of DM as an independent predictor of PH in chronic respiratory disease patients. The presence of DM indicates increased risk of PH in these patients.
